# Quinolizidine-Based Variations and Antifungal Activity of Eight *Lupinus* Species Grown under Greenhouse Conditions

**DOI:** 10.3390/molecules27010305

**Published:** 2022-01-04

**Authors:** Willy Cely-Veloza, Diego Quiroga, Ericsson Coy-Barrera

**Affiliations:** Bioorganic Chemistry Laboratory, Facultad de Ciencias Básicas y Aplicadas, Universidad Militar Nueva Granada, Cajica 250247, Colombia; diego.quiroga@unimilitar.edu.co

**Keywords:** Fabaceae, *Lupinus*, quinolizidines, alkaloids, *Fusarium oxysporum*, antifungals

## Abstract

*Fusarium oxysporum* is an aggressive phytopathogen that affects various plant species, resulting in extensive local and global economic losses. Therefore, the search for competent alternatives is a constant pursuit. Quinolizidine alkaloids (QA) are naturally occurring compounds with diverse biological activities. The structural diversity of quinolizidines is mainly contributed by species of the family Fabaceae, particularly the genus *Lupinus*. This quinolizidine-based chemo diversity can be explored to find antifungals and even mixtures to address concomitant effects on *F. oxysporum.* Thus, the antifungal activity of quinolizidine-rich extracts (QREs) from the leaves of eight greenhouse-propagated *Lupinus* species was evaluated to outline promising QA mixtures against *F. oxysporum.* Thirteen main compounds were identified and quantified using an external standard. Quantitative analysis revealed different contents per quinolizidine depending on the *Lupinus* plant, ranging from 0.003 to 32.8 mg/g fresh leaves. Bioautography showed that all extracts were active at the maximum concentration (5 µg/µL). They also exhibited >50% mycelium growth inhibition. All QREs were fungistatic except for the fungicidal QRE of *L. polyphyllus* Lindl. Angustifoline, matrine, 13α-hydroxylupanine, and 17-oxolupanine were ranked to act jointly against the phytopathogen. Our findings constitute reference information to better understand the antifungal activity of naturally afforded QA mixtures from these globally important plants.

## 1. Introduction

Fabaceae is the second largest family of flowering plants, containing approximately 490 species that have been used for medicinal purposes [1]. These plants are distributed in more than 65 genera of Fabaceae and can produce specialized metabolites such as quinolizidine alkaloid (QAs) (specially Genisteae Tribe) and non-nitrogenous compounds [2,3]. One of these genera is *Lupinus*, which is distributed in the Mediterranean, North and East Africa, and North and South America, and hosts the largest number of species [4]. About 170 *Lupinus* species can be found worldwide, including wild and domestic plants, widely called lupins [5]. In Colombia, 47 *Lupinus* species have been reported in the central and eastern mountain ranges in the Colombian Massif and Sierra Nevada de Santa Marta [6]. *Lupinus*-based products, particularly lupin seed proteins, are becoming good alternatives for multiple foods, including beverages, bakery, imitation meat, and dairy products, which have promoted intensive lupin cultivation in several parts of the world to produce grain protein [7]. However, the presence of QAs in lupin seed-based products provides a bitter taste. Therefore, debittering procedures should be performed on lupin seeds containing high QA contents for human and animal consumption [8].

*Lupinus* is one of the genera of Fabaceae that shows more considerable variability in QAs, which are biosynthesized by the *L*-lysine pathway in aerial green tissues, particularly leaves, and translocated to all plant parts whose accumulation is mainly in seeds (4–8% dry weight) [9]. Above 170 QAs have been detected, identified, and quantified in different *Lupinus* species from which bicyclic-, tricyclic-, and tetracyclic-type QAs can be obtained [10,11,12,13] and whose structural pattern is highly variable depending on the species [14]. The QA biosynthesis is highly regulated, influenced by genetic (e.g., genotype), biotic (e.g., presence of phytophagous insects and/or microbial pathogens), and abiotic (e.g., environmental and soil conditions) factors [13,14].

Lupin QAs constitute a natural defensive chemical barrier facing biotic pressures [15]. Thus, despite their well-known acute anticholinergic toxicity, they can be explored as alternatives (or even compound templates) against pests and pathogens. In this regard, some QAs isolated from *Lupinus*, such as sparteine, lupanine, and lupinine, have been used to control intestinal ectoparasites in cattle [16], and farmers occasionally use a QA-rich infusion from seeds for pest control in plants [17]. In addition, QA-rich *Lupinus* extracts (QREs) have shown antimicrobial activity against different microorganisms. For instance, the extracts of *L. mutabilis* seeds have inhibited 95% of the growth of *Staphylococcus aureus* [18], while Candida fungi have shown >60% inhibition in the presence of QREs of *L. arboreus* leaves [19], *L. albescens* [20], *L. angustifolius* [21], and *L. densiflorus* [22]. The seed-derived QREs from *L. exaltatus* and *L. mexicanus* have also been reported, showing inhibition >72% against *Sclerotium rolfsii*, *Alternaria solani*, *Rhizoctonia solani*, and *F. oxysporum* [23]. In the case of phytopathogens of the genus Fusarium, *F. oxysporum* and *F. verticillioides* showed susceptibility to QREs of *L. albescens*, with IC_50_ values < 30 μg/mL [20].

The current phytosanitary problems associated with the attack of *F. oxysporum* remain to be solved. Crops infected with this phytopathogen experience fusariosis, a plant disease that causes damage from leaf chlorosis to vascular necrosis [24]. These adverse effects produce significant economic losses in the agricultural sector [25], specifically in crops such as cape gooseberrys [26], bananas [27], carnations [28], lulos, and strawberries [29]. Fungicides such as dithane-M45, manzate 200WP, fitoraz, rovral-Flo, folicur EW, stroby SC, azoxystrobin, benomyl, carbendazim, and fludioxonil are currently used for the management of fusariosis [30,31]. However, the excessive use of these products led to adverse effects such as the production of resistant strains that seriously affect some important crops, such as bananas [32].

Similarly, other problems related to the inappropriate use of fungicides have affected the environment, microbiota, and soil fertility due to non-biodegradable, persistent, and bioaccumulative chemicals [33,34]. Therefore, there is a continual need to search for competent alternatives to synthetic pesticides to control this phytopathogen that can eventually be incorporated into rigorously controlled integrated pest management (IPM) programs. The exploration to find antifungals might be advantageous from plant sources with favorable propagation, such as species of the genus *Lupinus*. In addition, since the particular production mainly contributes to the structural diversity in *Lupinus* leaves and considering that QAs are biologically active specialized metabolites and plant defense products [2], naturally-supplied QA mixtures could be examined as a promising approach for exerting concomitant effects on *F. oxysporum*.

Therefore, the present study evaluated the antifungal activity against *F. oxysporum* of QREs obtained from eight *Lupinus* species cultivated under greenhouse conditions. Thus, the quinolizidine variations in the test *Lupinus* species were employed to recognize patterns and identify the most active QAs and their mixtures as plausible antifungals from foliar materials of cultivated lupin plants.

## 2. Results and Discussion

### 2.1. Characteristics of Propagated Plants

The leaf collection stage of native species was defined when plants showed their first flower buds and reached ca. 50 cm in height. Only average heights around 20 cm were reached in exotic species, except *L. albus* specimens that exceeded 50 cm in height. In the case of *L. polyphyllus* and *L. perennis*, no floral buds were observed. The flowering could be affected by the unusual temperature conditions of the greenhouse (mainly cold periods and irradiation) for these exotic plants, since vernalization and photoperiods have been described to affect the flowering periods of some lupins seriously, e.g., *L. luteus* [35].

Additionally, high temperatures can alter the phenological stages of *Lupinus* [36] in such a way that lower production of flowers and seeds is observed, while in more extreme cases, the flowering stage is not reached [37]. However, exotic lupin plants grew under the employed propagation conditions, indicating good acclimatization of *Lupinus* species to such a growing environment, which is beneficial when mass cultivating for harvesting purposes. Thus, all lupin plants reached satisfactory growth to collect enough leaf biomass for the subsequent chemical analysis.

### 2.2. Chemical Analysis

The young leaves were collected once the lupin plants reached physiological maturity (i.e., 70 ± 19 days after transplantation). Hence, QREs from fresh leaves (5 g) of the eight *Lupinus* species were prepared to afford the yields shown in Table S1 (supplementary material), following an acid-base extraction method. The highest yields were achieved for *L. bogotensis*, *L. mirabilis*, and *L. mutabilis* (yields > 5%). Contrarily, the exotic species yielded lower extract amounts, but the best yields between them resulted for *L. polyphyllus*, *L. perennis,* and *L. albus* (yields > 4%), while *L. argenteus* and *L. arboreus* showed the lowest yields (<3%). The yield differences of native lupine plants compared to exotic ones can be explained by biomass and specialized metabolite variations due to those intrinsic (genetic) factors favored by extrinsic (environmental) factors related to the propagation conditions managed in the present study [38].

GC-MS analysis subsequently recorded the QA composition of these QREs obtained from the eight *Lupinus* plants. With the diagnostic analysis of the mass spectra combined with the retention indices compared to the available information in literature, QAs **1**–**13**, which are listed in Table 1 and whose structures are shown in Figure S1, were identified.

**Figure 1:**
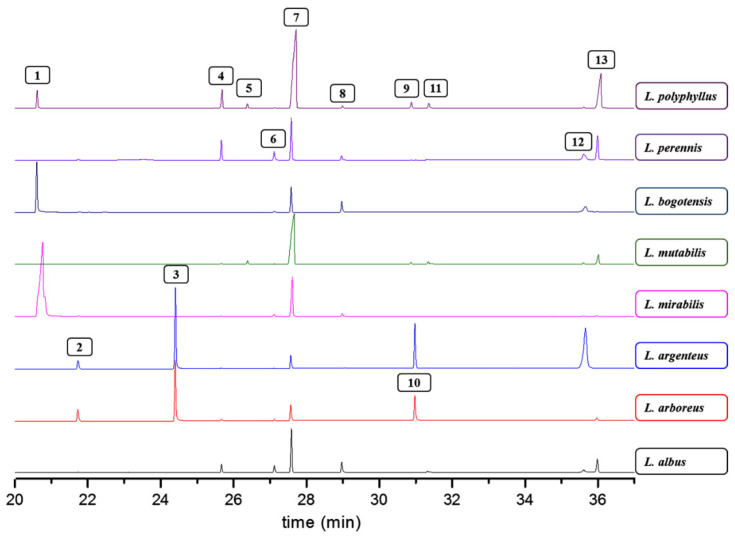
Stacked GC-MS chromatograms of quinolizidine-rich extracts (QREs) were obtained from the eight species of *Lupinus*. The box-enclosed numbers represent each identified quinolizidine listed in Table 1.

The identified QAs have already been reported as phytocomponents in *Lupinus* species. Some of them are commonly found as the most abundant QAs, being part of certain mixtures at differentiated proportions between species or even due to the effects caused by biotic and abiotic factors [13,44]. Qualitative and quantitative comparisons were made between the eight lupin species propagated under the same conditions in the present study. Accordingly, the stacked chromatographic profiles are shown in Figure 1 and the measured contents per QA of these test plants, expressed as mg/g fresh leaves (fl), are presented in Table 2. Some compound signals (i.e., **6**) are not entirely visualized in Figure 1 due to the relative abundance, despite being quantified according to the limit of quantification (see Section 3.3), since the most abundant compounds per GC-MS chromatogram reduced the visible intensity of those signals related to the minor compounds.

The QRE profiles changed in the presence and abundance of QAs, whose contents varied within a 0.031–32.8 mg/g fl range. Some common QAs were found in all test species, as in the case of **6** and **7**, with contents between 0.046–3.12 and 5.62–25.7 mg/g fl, respectively. In contrast, other QAs were not commonly found in the sample set, such as **9** (0.043–3.54 mg/g fl), as most reported in the genus Sophora [43]. In the case of the species *L. argenteus* and *L. arboreus*, QAs containing the 2-pyridone moiety, such as **3** and **10**, were the most common compounds, comprising contents between 4.61 and 6.08 mg/g fl. A previous study reported that *L. argenteus* ecotypes exhibited the presence of seventeen QAs, including lupanine derivatives such as 5,6-dehydrolupanine, α-isolupanine, 11,12-dehydrolupanine, 3-hydroxylupanine, and thermopsine [45], but the abundance and presence of lupanine derivatives were found to be different in *L. argenteus* under study. QAs **7** and **11** have been previously reported for *L. arboreus*, but **11** mainly accumulates in *L. arboreus* seeds; therefore, it was not detected in this study [46]. In the case of *L. albus*, QAs **4**, **7**, **8**, and **12** were identified and quantified (between 0.91 and 13.8 mg/g fl). However, the presence of albine was reported in previous studies [47], but it was not detected in the *L. albus*-derived extracts under investigation. Finally, in native species such as *L. mutabilis* and *L. bogotensis*, compounds **1**, **2**, **4**–**8**, and **12** have previously been reported [8,48], agreeing with the results of the present study involving the highly-varied contents of such QAs (between 0.033 and 32.8 mg/g fl). The presence of **1**, **7**, **9**, and **10** has been generally reported in the genera *Lupinus*, *Sophora*, and *Genista* species.

All *Lupinus* species were characterized by the high production and accumulation of lupanine (**7**), exploited as a useful chemotaxonomic marker to recognize *Lupinus* species [2]. In native species, close lupanine contents were found, although *L. mutabilis* exhibited the highest and significantly different content. In the case of exotic species, some differences were found and could be gathered into two groups as follows: In the first group, *L. argenteus* and *L. arboreus* were characterized by the low content of **7** (<7 mg/g fl) and the presence of QAs **3** and **10**, which are not commonly reported in *Lupinus* species but highly occurred in Genista species [49,50]. In the second group, the species *L. polyphyllus*, *L. albus*, and *L. perennis* showed important contents of **7** but the highest abundance of **4** (5.39, 3.35, and 3.12 mg/g fl, respectively) and other characteristic compounds of these species such as **8** and **11** (within content ranges of 0.735–6.15 and 0.068–2.62 mg/g fl, respectively).

### 2.3. Direct Bioautography Assay

**Figure 2:**
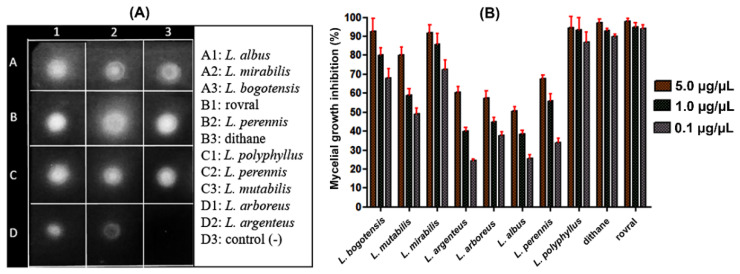
Antifungal activity against *F. oxysporum* QREs from eight lupin species. (**A**) Bioautography results using 50 µg of QREs; (**B**) Mycelial growth inhibition percentages of eight QREs at three concentrations.

Bioautography allowed qualitatively observing the sensitivity of the phytopathogen spores to the test QREs (Figure 2A). In this way, the negative control (D3, distilled water) exhibited a completely dark surface, representing the phytopathogen growth in that square [51]. In contrast, positive controls Dithane and Rovral (squares B1 and B3) revealed clear zones, indicating no fungal growth due to the inhibition of spore germination [52]. Similarly, QRE-based treatments (squares A1–A3, B2, C1–C3, and D1–D2) produced similar inhibition halos to positive controls. Thus, although some distinctive intensities were evidenced, these results qualitatively indicated that all test extracts exhibit antifungal activity on *F. oxysporum* spores.

### 2.4. Mycelial Growth Inhibition Assay

The antifungal activity evaluation of the *Lupinus*-derived QREs against *F. oxysporum* was quantitatively extended by assessing the mycelium growth inhibition through the amended-medium (poisoned food) technique. Thus, QREs were evaluated at three doses (i.e., 5.0, 1.0, and 0.1 µg/µL) and, subsequently, the respective inhibition percentages were measured, obtaining values >50% at the maximum concentration (Figure 2B). The *L. polyphyllus*-derived QRE showed the best antifungal activity at the three test concentrations (inhibition > 85%), and such inhibitions were very similar to those of positive controls. However, the observed antifungal activity variations depended on the treatment and QAs composition involved significant differences (*p* < 0.05). Thus, using the post hoc Tukey test, the statistically significant differences based on variations in composition and concentration were obtained. Consequently, twenty different mean groups were achieved (Figure S2, supplementary material). The first significantly different groups (i.e., A–F) were related to the best inhibition values (>80%), comprising positive controls and extracts from *L. polyphyllus*, *L. bogotensis*, *L. mirabilis*, and *L. mutabilis* at the highest test concentrations (1 and 5 µg/µL). The other groups represented a lesser inhibition (<75%) showed by the other extracts, mainly dependent on the concentration of each QRE. The measured inhibition values agree with the values reported for QREs obtained from *L. exaltatus* [53]. *L. argenteus*, *L. arboreus*, *L. albus,* and *L. perennis* exhibited the lowest inhibitory activity on mycelial growth (inhibition between 20 and 60%, depending on the concentration). However, *L. albus* and *L. perennis* exhibited clear zones of inhibition in the bioautography, even similar to other QREs treatments (Figure 2A), suggesting a plausible selective effect depending on the fungal structure (spore > mycelium), which might be clarified in further studies.

**Figure 3:**
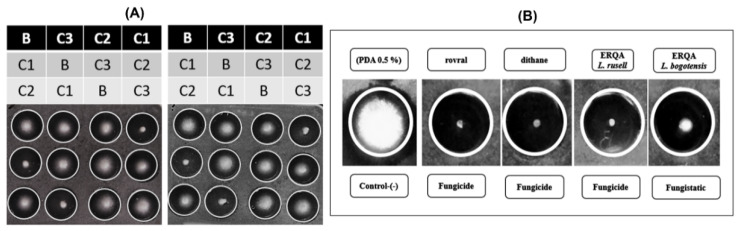
(**A**) Antifungal activity (mycelial growth inhibition) evaluated for the most-active QREs from *L. polyphyllus* (**left**) and *L. bogotensis* (**right**) against *F. oxysporum*. (**B**) (PDA 0.5%), **C1** (5 µg/µL), **C2** (1 µg/µL), and **C3** (0.1 µg/µL). (**B**) Fungicide vs. Fungistatic of the most-active QREs of *L. polyphyllus* and *L. bogotensis*.

The test QREs showed differences in their chemical composition and antifungal activity against *F. oxysporum*. In general, the eight lupin species showed the best antifungal activity at the maximum test concentration (5 µg/µL), comprising inhibition percentages between 50–95%. As mentioned above, the most active QREs were those obtained from *L. polyphyllus* and *L. bogotensis*. These QREs showed dose-dependent differences, as shown in Figure 3A. For example, **C1** (treatment at 5 µg/µL) highly inhibited the mycelial growth of the phytopathogen (inhibition > 95%), while **C2** (treatment at 1 µg/µL) showed lower mycelial growth (inhibition > 80%). In the case of **C3** (treatment at 0.1 µg/µL), moderate mycelial growth was observed (inhibition > 70%). On the other hand, the intermediate doses (**C2**) of the QREs obtained from *L. polyphyllus*, *L. bogotensis*, and *L. mirabilis* showed the best results (inhibition > 80%), while a significant inhibitory reduction was found for the other five species (inhibition < 60%). Finally, the least active concentration was **C3**. At this dose, *L. polyphyllus*, *L. bogotensis*, and *L. mirabilis* afforded the best results (inhibition > 70%), while QREs from the other species were found to be below 60%. In this sense, these results suggest that the optimal threshold for an effective dose to inhibit the growth of *F. oxysporum* mycelium by these QREs is 5 µg/µL (0.5% *w*/*v*).

Once the mycelial growth inhibition assay time elapsed, a subsequent test was performed to classify the extracts as fungicidal or fungistatic. This test was accomplished by further monitoring the fungal growth in fresh PDA after completion of QRE treatment at 5 µg/µL. This test suggested that seven lupin-derived extracts were fungistatic (Figure 3B). In contrast, there was no mycelium growth for the *L. polyphyllus*-derived QRE, indicating a fungicidal behavior. In addition, there are some studies related to QAs and bisquinolizidine alkaloids, such as spirocytisine, 3,5-dibromocytisine, bromo-*N*-boccytisine, *N*-boccytisine, 3-bromobenzyl cytisine, 4-bromo-benzylcytisine, 3-iodobenzylcytisine, and 4-iodobenzylcytisine, evaluated against *Aspergillus niger* van Tieghem ATCC 6275, and all of them showed fungistatic activity [54].

**Figure 4:**
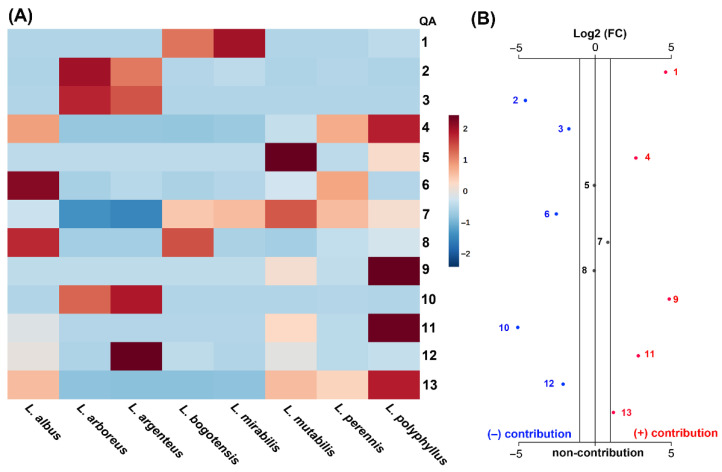
Comparison of QA contents (mg/g fresh leaves) among QREs obtained from leaves of eight lupin species. (**A**) Heatmap visualization of the normalized content distribution (unit of variance scaling) of QAs **1**–**13** from test lupins. Autoscaled QA contents are related to the color scale: 2 = high content; −2 = low content. (**B**) Unpaired fold-change (FC) analysis plot for selecting important QAs with threshold 2, by integrating the chemical (QA contents) and biological (mycelial growth inhibition) data. Antifungal activity was used as a categorical variable, subdividing the antifungal activity of QREs into two datasets: more active (>80% inhibition) and less active (<75% inhibition), according to the Tukey test, to supervise the QA selection. According to the antifungal activity, positive and negative contributions to the FC-based QA selection are depicted as red and blue numbers and dots.

The detailed scrutiny of the chemical and antifungal activity data integration of test QREs led us to recognize meaningful patterns. Initially, intuitive visualization of the comparison of autoscaled QA contents revealed plausible contributions by some QAs to the antifungal activity (Figure 4A). In this regard, QREs containing particular combinations of specific QAs were generally the most active extracts. Thus, the most active QRE, derived from *L. polyphyllus*, exhibited the abundant presence of **4**, **9**, **11**, whereas QREs of *L. bogotensis* (the second most active extract against *F. oxysporum*) contained **1** and **8** in high abundance and *L. mirabilis* exhibited the highest content of **1**. Remarkably, compound **9** was only detected in the most active QREs from *L. mutabilis* and *L. polyphyllus*, whose interesting inhibitory properties on conidia germination of *F. oxysporum*, *Sphaeropsis spainea*, *Valsa pini* (IC_50_ < 600 µg/mL) were previously evaluated [55]. The other lupin species that did not contain **1**, **4**, **9,** and/or **11,** showed the lowest activity, with inhibition < 70% in the three treatments. This in-depth exploration was extended by an unpaired fold-change (FC) analysis performed on QA content data of the test QREs, involving two antifungal activity-related datasets, i.e., more active QREs with >80% inhibition and less active QREs with <75% inhibition. The resulting plot (Figure 4B) showed the important QAs selected by this FC analysis (threshold = 2), and the red and blue dots represent QAs above and below such a threshold, respectively. This logarithmic relative change between the two activity-related datasets can be statistically interpreted as a positive and negative contribution to the antifungal activity by a QA due to its particular presence in a more active or less active QRE, respectively. Thus, QAs **2**, **3**, **6**, **10**, and **12** contributed negatively to the antifungal activity, whereas **5**, **7,** and **8** exhibited no contribution since the FC threshold was not exceeded. In contrast, the significance of **1**, **4**, **9**, **11**, and **13** was statistically evidenced by their positive contribution to the antifungal activity. They can be considered essential constituents of a bioactive QRE and a plausible, promising mixture against *F. oxysporum*. The particular combination of QAs in the *L. polyphyllus*-derived QRE might rationalize its observed fungicidal activity. In this regard, such a QA-based combination could be due to the growing behavior at the test greenhouse conditions since this introduced plant did not reach the flowering stage. This fact may be a critical factor influencing the composition and, consequently, the antifungal activity of this most active lupin plant.

On the other hand, this integrated examination also suggests that very high contents of **7** are not a determinant for antifungal activity against *F. oxysporum* as the five selected QAs. This observation agrees with a previous study on a QRE obtained from *L. exaltatus* seeds. Such an extract did not show antifungal activity against *F. oxysporum* and its QA composition was based on a high relative abundance of **7** (53.2%), along with other derivatives and aphylline and sparteine in lower relative abundance (<10%) [53]. Therefore, a QRE whose composition mainly contains compound **7** and some of its analogs, such as *L. albus*-derived QRE, cannot be considered effective in inhibiting the *F. oxysporum* growth (<50%). A similar result was found in a previous study with a QRE obtained from *L. mexicanus* seeds, whose practically insignificant antifungal activity against *F. oxysporum* was observed. The test extract was mainly composed of high contents of **7** (content = 21.2 mg/g, relative abundance = 76.2%), along with other QAs with lower content (<3 mg/g) and relative abundance (<10%) [56]. Finally, the activity of QREs having good contents of **3** and **10** (4.61–6.08 mg/g fl) was found to be moderate to low. In this regard, other QAs containing the 2-pyridone moiety (e.g., cytisine) have exhibited bactericidal and antifungal activities [54,57]. However, the antifungal activity of **3** and **10** could not be statistically recognized, possibly by low inhibition by structural reasons that should be deepened in further structure-activity relationship studies.

### 2.5. Variation in the QA Profiles of L. polyphyllus Induced by Pruning Events

*L. polyphyllus* “Russell” was an exotic lupin species that quickly propagated under these greenhouse conditions, although a flowering stage was not reached. However, the leaf production was abundant, so periodically (every two weeks), the young leaves were pruned to obtain 5 g of fresh plant material. This pruning process was performed twelve times, and consequently, twelve QREs were obtained which were further analyzed by GC-MS. This pruning-based exploration on this most active QRE-producing lupin plant helped refine the chemical and biological data integration to select bioactive QAs through inherent variations.

**Figure 5:**
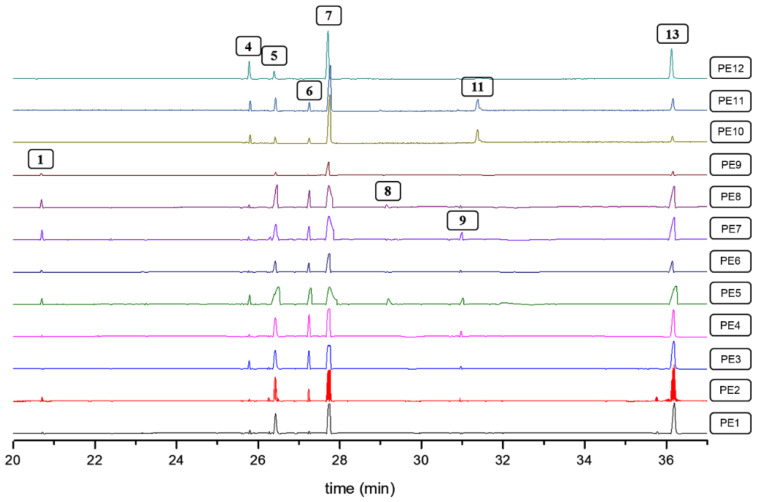
Stacked GC-MS chromatograms of quinolizidine-rich extract (QRE), obtained after pruning events (PE1–12) on *L. polyphyllus*. The box-enclosed numbers represent each identified quinolizidines, listed in Table 1. PE = pruning event.

The resulting chromatograms are shown in Figure 5. The QREs of *L. polyphyllus* were qualitatively and quantitatively compared, and some changes associated with the presence and abundance of detected alkaloids in each resulting QRE were observed. Hence, ten QAs were detected and quantified (contents between 0.003 and 1.86 mg/g fl), along with pruning events (PEs) (Table 3).

In general, all QREs contained **4**, **5**, **7**, **13**, while **1**, **6**, **8**, **9**, **11**, **12** were evidenced in selected PEs. Notably, compound **1** showed content variations for each PE (0.009–0.375 mg/g fl), with PE7-8 showing significantly higher abundance. Compound **1** has been previously reported in *Lupinus* genotypes, and its antimicrobial history against *K. pneumoniae* and *P. aeruginosa* has been highlighted [18]. A content increase for **9** was observed from PE3-7, but no longer detected from PE9. This compound is vital due to its antimicrobial [55,58], antiparasitic [59], cytotoxic [60], anticarcinogenic [61], antiviral, [62] and antimalarial properties [63,64].

**Figure 6:**
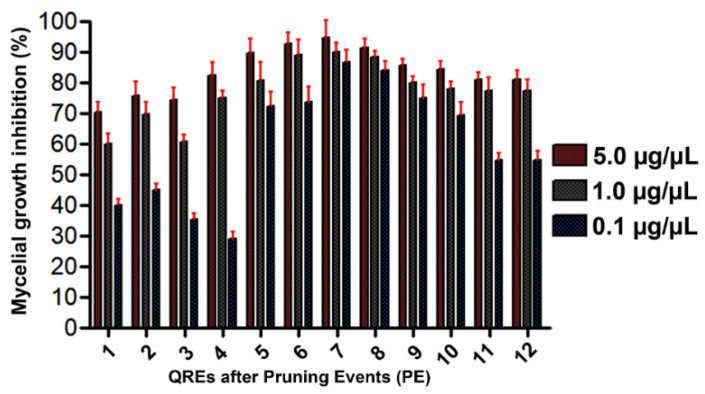
Mycelial growth inhibition percentages of QREs at three concentrations after pruning events (PE1–12) on *L. polyphyllus*.

**Figure 7:**
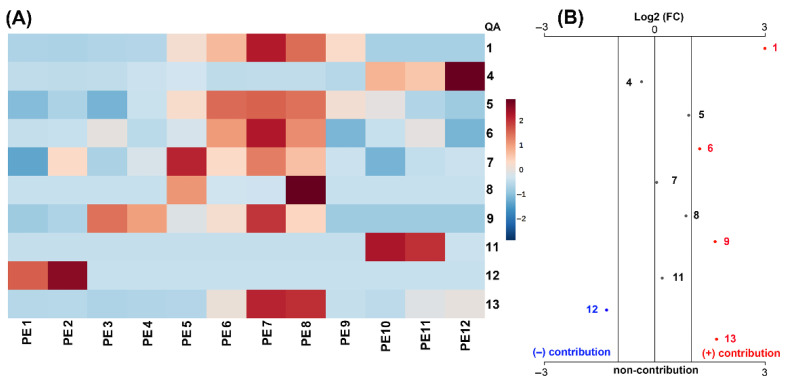
Comparison of quinolizidine alkaloid (QA) contents (mg/g fresh leaves) of quinolizidine-rich extracts (QREs) obtained after twelve pruning events on *L. polyphyllus*. (**A**) Heatmap visualization of the normalized content distribution (unit of variance scaling) of quantified QAs of each resulting QRE. Autoscaled QA contents are related to the color scale: 2 = high content; −2 = low content. (**B**) Unpaired fold-change (FC) analysis plot for selecting important QAs with threshold 2, by integrating the chemical (QA contents) and biological (mycelial growth inhibition) data. Antifungal activity was used as a categorical variable, subdividing the antifungal activity of QREs into two datasets: more active (>90% inhibition) and less active (<85% inhibition), according to the Tukey test, to supervise the QA selection among QREs. According to the antifungal activity, positive and negative contributions to the FC-based QA selection are depicted as red and blue numbers and dots.

The QREs obtained from the twelve PE on *L. polyphyllus* were evaluated through mycelium growth inhibition tests at 5, 1, and 0.1 µg/µL (Figure 6). In general, the QREs from PE1–4 corresponded to mixtures that contained low amounts (<14 mg/g fl) of compounds structurally related to **7,** and whose inhibition was <80%, while PE5-9 exhibited the combined high presence of **1**, **9** and **13** (Figure 7A) which seemed to be responsible for the best activity, especially in PE7–8, whose inhibition was >85%. This fact is possible since these compounds were previously reported to exhibit antifungal activity against pathogens like *Valsa pini*, *Cladosporium oxysporum*, *Sphaeropsis sapinea*, *Marssonina brunne*, *A. solani*, *M. fructicola*, and *F. oxysporum* [55,57]. There was no substantial decrease in antifungal activity between PE10–12 and PE5–9, despite the absence of **1** and **9**. However, the presence of **11**, which is a hydroxylated derivative of **7**, has also been reported to have antimicrobial activity against *E. coli*, *P. aeruginosa*, *B. subtilis*, *S. aureus*, *C. albicans*, and *C. krusei* [21].

The unpaired FC analysis of the PE1–12-derived QREs showed that **1**, **6**, **9**, and **13** contributed positively to the antifungal activity (Figure 7B), and the extracts containing these selected QAs would be promising mixtures to inhibit the growth of *F. oxysporum* under in vitro conditions, within the pruning-dependent natural supply of QA from *L. polyphyllus*. Hence, such mixtures and other variations for phytopathogen management and control at different scales can be explored.

There is a relevant concern about QA-rich lupin-based foods for human and animal consumption related to the acute anticholinergic toxicity of QAs, which encouraged several procedures for seed debittering and the development of lupin varieties that produce/accumulate low QA amounts [13,65]. Additionally, the QAs structurally related to **10** (i.e., 2-pyridone-containing tetracyclic QAs) have shown a teratogenic background. They are responsible for Holstein cattle’s so-called twisted calf disease [66]. Therefore, although lupin QAs are recognized as natural defensive compounds (Wink, 2018), and this feature can be exploited for pest management, their development as chemical agents for controlling phytopathogens should be rationally and carefully conducted. In this regard, these findings can serve as a reference in future studies to explore the composition-activity (and even structure-activity) relationship of QAs to optimize such an antifungal potential and reduce possible side effects due to the toxicity.

## 3. Materials and Methods

### 3.1. Propagation of Lupinus Plants under Greenhouse Conditions

Seeds of the three native species, i.e., *L. bogotensis* Benth., *L. mutabilis* Sweet, and *L. mirabilis* C.P. Sm., were collected on the campus at Military University Nueva Granada (UMNG) (4°56′ N, 74°00′ W and 2562 masl). Seeds of five exotic species, i.e., *L. albus* L., *L. argenteus* Pursh, *L. polyphyllus* Lindl. “Russell”, *L. perennis* L., and *L. arboreus* Sims, were commercially purchased from Sow Right Seeds (https://sowrightseeds.com, accessed on 30 November 2021).

All the *Lupinus* seeds were superficially disinfected with 70% ethanol (2 min) and then with 3% sodium hypochlorite (5 min), subsequently washed with sterile distilled water (3 × 3 min), and cleaned with a drop of Tween 20. The seeds were planted in 72-cell seedbeds that contained a substrate of loamy-silty soil (LSS) with rice husk (RiH) at a 3:1 ratio and were maintained under greenhouse conditions (temperature = 21 ± 4 °C; relative humidity (RH) = 65 ± 15%, altitude = 2562 masl, total light transmission = 85 ± 5%, total light diffusion = 55 ± 5%, and UV transmission between 290–340 nm = 5%) during the whole propagation experiment (30 days). At 8–10 days after seed planting (dasp), the seeds started the germination process (i.e., seed coat breakdown) and subsequent embryonic root (radicle) growing. Cotyledons appeared between 12–15 dasp on average.

After 20–30 dasp, the first and second pairs of leaves emerged, and the resulting seedlings (*n* = 20) were transplanted in 2 L bags on a substrate containing a mixture of LSS/RiH 3:1 and maintained under the same greenhouse conditions. After transplanting, the seedlings were watered with water (500 mL) every two days, and once the elongation of the central axis began to be observed, we proceeded to apply commercial triple-15 fertilizer (5%). After 70 ± 19 days of development under these conditions (i.e., after transplanting) to reach the flowering stage, fresh leaves were collected for QA extraction.

In addition, a new set of plants of *L. polyphyllus* were propagated at the same greenhouse conditions. After 50 days of transplanting, leaves were removed every two weeks from the same living, growing plants (*n* = 10) and subsequently prepared for QA extraction. Each pruning event (PE) was performed at the same time of the day (i.e., 10 a.m.). This pruning process was repeated twelve times (PE1–PE12).

### 3.2. Preparation of Quinolizidine-Rich Extracts (QREs)

Fresh young leaves (5 g) of each biological replicate (*n* = 10) were extracted with 0.5 M HCl (20 mL) under stirring for 24 h at 130 rpm in an orbital shaker. Subsequently, the acidic solution was filtered and alkalinized to pH = 10 with a 15% aqueous NH_3_ solution_._ Subsequently, liquid-liquid extraction was performed using chloroform to obtain an organic phase enriched in QAs. Finally, the solvent was removed by distillation under reduced pressure at 375 mbar for 5 min, and *Lupinus* QREs were then obtained.

### 3.3. Gas Chromatography Coupled to Mass Spectrometry (GC-MS)

The chromatographic profiles and mass spectra were obtained with a Thermo Trace 1300 equipped with a flame ionization detector (FID) and coupled to an ISQ LT mass spectrometer with a single quadrupole analyzer. For the analysis, an Rxi^®^ 5Sil MS column (5% diphenyl/95% dimethylpolysiloxane, 60 m, 0.25 mm ID, and 0.25 μm) was used. A temperature program was implemented; the starting temperature was 120 °C, maintained for 2 min, and then a 6 °C/min program was applied until 300 °C and kept for 10 min. The test QREs were prepared at 1 μg/μL in CH_2_Cl_2_ (GC-MS grade, SupraSolv^®^). The injection volume was 1 μL in split mode (split ratio = 30). The transfer line temperature was 250 °C, and the carrier gas was grade-5 helium (flow = 1 mL/min). The ionization mode was the electronic impact (EI) at 70 eV. The retention indices (RI) were calculated using a series of C_10_-C_24_ *n*-alkanes [67], according to Equation (S1) (supplementary material). Compounds **1–13** were identified by diagnostic analysis of their mass spectra and comparison of RIs with available literature. On the other hand, the QAs quantification was performed using the external standard method by GC-FID under the same chromatographic conditions. The standard curve was built by injecting eight solutions (ranging from 1 to 500 µg/mL) of (+)-lupanine (100 µg/mL, Sigma-Aldrich, St. Louis, MO, USA). The instrument response was verified by adding caffeine (100 µg/mL, Sigma-Aldrich, St. Louis, MO, USA) as an internal standard. Owing to limitations related to commercial availability or purity degree of reference compounds of identified QAs compounds, **1**–**13** were quantified as lupanine equivalents and expressed as mg lupanine equivalents per gram of fresh leaves (mg LE/g fl). Relative response factors were used to correct the peak areas of detected QAs. Quantitative analysis was performed in triplicate. The intra and inter-day analyzes of (+)-lupanine were used to evaluate the method precision, whose relative standard deviations (RSD %) were 2.4 and 3.7%, respectively. The limit of detection (LOD) and limit of quantification (LOQ) of (+)-lupanine was 1 and 2 µg/mL, respectively. Finally, the GC-MS-derived data were processed in MZmine 2 software to compare the resulting chromatographic profiles of QREs. Thus, the baseline correction was performed and exported as total ionic current (TIC) to a CSV file. Data were normalized and autoscaled. The resulting autoscaled profiles were stacked in OriginPro 8.5 for intuitive visualization of the QA variations between each QRE.

### 3.4. Direct Bioautography Assay

A spore suspension of the fungus *F. oxysporum* was prepared at a concentration of 1 × 10^6^ CFU/mL on a nutrient broth containing KH_2_PO_4_ (7 g), Na_2_HPO_4_.2H_2_O (3 g), KNO_3_ (4 g), MgSO_4_.7H_2_O (1 g), and NaCl (1 g) per liter of water. The culture broth was sterilized for 1 h in an autoclave at 120 °C. Then the spore suspension was prepared, adding 30% glucose (10 mL) for every 60 mL standard solution. Respective QRE solutions were prepared in CHCl_3_ at 1 mg/mL. Subsequently, the QRE solutions (50 µL) were seeded on silica gel 60 F_254_ thin-layer chromatography (TLC) plates (20 × 20 cm, 0.20 mm layer thickness, subdivided into squares), and the solvent was removed in an extraction chamber. Then, the spore solution of *F. oxysporum* was sprayed on the plate surface and placed into a humid and dark chamber at 25 °C for 72 h. Dithane (mancozeb) and Rovral (iprodione) were used as positive controls at the same doses. In this test, the antifungal activity was determined by light areas on the squares that indicated no fungal growth by inhibiting spore germination, contrasted with dark areas that revealed fungal growth [68].

### 3.5. Mycelial Growth Inhibition Assay

Antifungal activity evaluation of QREs was performed by measuring the growth halo of the phytopathogen *F. oxysporum* with the presence of the extracts at different concentrations compared to that of a blank (0.5% PDA), using the amended-medium procedure [69]. The culture medium contained 2.4% PDB and 1.5% bacteriological agar in 100 mL of distilled water. The medium was homogenized for 2 min in a microwave oven and then sterilized in an autoclave for 1 h at 120 °C. The culture medium (20 mL) was then placed into a previously sterilized Petri dish to propagate the fungus. Once it cooled and solidified, a 2-mm plug from a previously prepared monosporic culture was placed onto the central part of the Petri dish and left to grow at 28 °C for 8 days.

Three treatments per QRE were prepared for the antifungal assays according to three final QRE concentrations (i.e., 5, 1, and 0.1 µg/µL). Thus, the required amount of the respective QRE was dispersed in 0.5% PDA to afford the QRE-amended medium per treatment. Subsequently, each treatment was randomly placed in a 12-well glass plate (79 × 63 × 4 mm). Finally, a 1.1-mm plug (equivalent to the internal diameter of a 1.1-mm borosilicate capillary tube) was taken from an 8-day phytopathogen culture and placed onto the center of each well (QRE-amended and non-amended). This plate was placed into a humid chamber for 72 h at 25 °C. The evaluation of each concentration per QRE treatment was performed in triplicate. Dithane (mancozeb) and Rovral (iprodione) were used as positive controls at the same doses. After the incubation time, a photograph of the 12-well plate was taken and analyzed in ImageJ software, whose growth areas of control (non-amended) and QRE-amended wells were measured. The comparison of these areas led to the determination of the inhibition percentage using Equation (1).

(1) $$\mathrm{Inhibition}\;\mathrm{Percentage}=\frac{{\mathrm{area}}_{\mathrm{control}}-{\mathrm{area}}_{\mathrm{QRE}}}{{\mathrm{area}}_{\mathrm{control}}}\times 100\%$$

### 3.6. Fungicidal (FC) and Fungistatic (FS) Activity

For the fungicidal or fungistatic activity classification procedure, the central plug of the phytopathogen used in the prior 5-µg/µL-amended treatment was retrieved and placed onto fresh, non-amended PDA medium for 72 h. After this time, mycelial growth was additionally monitored. The QRE was classified as fungistatic or fungicidal if mycelial growth or no mycelial growth, respectively, was observed [70].

### 3.7. Data Analysis

A Shapiro-Wilks normality test was accomplished to examine the normal distribution of the quantitative data (*p* > 0.05). Once the normal distribution of the data was verified, an analysis of variance (ANOVA) was subsequently performed, followed by a post hoc Tukey test to establish significant differences between samples (*p* < 0.05). These analyses were performed in Infostat statistical software [71]. In addition, a heatmap was constructed using normalized QA contents through autoscaling. The antifungal activity was subsequently divided into two datasets (i.e., more and less active) depending on the significantly different mean groups after the Tukey test. An unpaired fold-change analysis (threshold = 2) was finally performed on chemical and antifungal activity datasets to select important QAs that positively and negatively contribute to mycelial growth inhibition. 

## 4. Conclusions

The present study attempts to combine the information of QA profiles and the antifungal activities against *F. oxysporum* of lupin derived QREs. This chemical and antifungal dataset integration provided valuable information of naturally afforded QA mixtures to understand the promising concomitant effects against *F. oxysporum*. In summary, we found that the test QREs showed important antifungal activity since all extracts showed inhibition > 50% at the maximum test concentration (5 µg/µL). The best antifungal results were obtained for the QREs of *L. mirabilis*, *L. polyphyllus* “Russell”, and *L. bogotensis*, reaching inhibitions ≥ 90% and similar to positive controls. In addition, all QREs were classified as fungistatic except for the *L. polyphyllus*-derived QRE ranked as a fungicide. QREs obtained after twelve pruning events on *L. polyphyllus* exhibited antifungal activity variations, with the QREs derived from PE7–8 being the most active extracts. Each test extract in the present study showed differential QA profiles. In-depth qualitative and quantitative analysis of such QA differences indicated that QREs containing particular combinations of angustifoline (**4**), α-isolupanine (**6**), matrine (**9**), 13α-hydroxylupanine (**11**), and 17-oxolupanine (**13**) best inhibit the growth of *F. oxysporum*. Contrarily, lupanine (**7**) seems to have a low contribution to the antifungal activity despite its high abundance in test QREs. A better understanding of such bioactivity of QA mixtures would promote further studies to deeply understand and exploit their antifungal potential at different levels (i.e., greenhouse and field conditions) for managing and controlling *F. oxysporum* within IPM programs.

## Figures and Tables

**Table 1:** Identified quinolizidines (**1**–**13**) in eight lupin species propagated under greenhouse conditions.

# ^a^	Rt ^b^ (min)	Name	RI ^c^	RI ^d^	Reference ^d^
1	20.5	sparteine	1785	1805	[39]
2	21.6	11,12-dehydrosparteine	1841	1841	[40]
3	24.4	*N*-methylcytisine	1918	1924	[41]
4	25.8	angustifoline	2079	2073	[39]
5	26.3	5,6-dehydrolupanine	2104	2092	[40]
6	27.1	α-isolupanine	2107	2123	[39]
7	27.7	lupanine	2170	2146	[42]
8	29.0	nuttalline	2338	2348	[18]
9	30.9	matrine	2366	2365	[43]
10	31.1	anagyrine	2390	2377	[40]
11	31.4	13α-hydroxylupanine	2405	2400	[39]
12	35.7	multiflorine	2440	2469	[18]
13	36.0	17-oxolupanine	2482	2473	[18]

^a^ Compound numbers according to the chromatographic elution presented in Figure 1; ^b^ Rt = retention time (min); ^c^ Calculated retention index (RI) according to the Equation (S1); and ^d^ Reported RI according to the respective reference.

**Table 2:** Contents of quinolizidines **1**–**13** in leaves of eight *Lupinus* species.

Plants	QA Content (mg LE/g FL) ^a^						
	1	2	3	4	5	6	7
*L.po.*	1.43 ± 0.12 ^C^	n.d.	n.d.	5.39 ± 0.18 ^A^	0.391 ± 0.008 ^B^	0.169 ± 0.009 ^E^	17.1 ± 0.9 ^C^
*L.pe.*	n.d.	0.031 ± 0.001 ^D^	n.d.	3.12 ± 0.12 ^B^	n.d.	1.607 ± 0.032 ^B^	20.0 ± 0.2 ^B^
*L.b.*	22.4 ± 0.7 ^B^	0.033 ± 0.002 ^D^	n.d.	n.d.	n.d.	0.083 ± 0.002 ^F^	19.3 ± 0.8 ^B^
*L.mu.*	n.d.	n.d.	n.d.	0.87 ± 0.09 ^C^	1.42 ± 0.16 ^A^	0.452 ± 0.009 ^C^	25.7 ± 0.6 ^A^
*L.mi.*	32.8 ± 0.6 ^A^	0.102 ± 0.004 ^C^	n.d.	0.102 ± 0.008 ^D^	n.d.	0.151 ± 0.006 ^E^	20.1 ± 0.31 ^B^
*L.arg.*	n.d.	1.38 ± 0.19 ^B^	4.98 ± 0.19 ^B^	0.051 ± 0.003 ^E^	n.d.	0.201 ± 0.016 ^D^	5.62 ± 0.14 ^F^
*L.arb*	n.d.	2.07 ± 0.16 ^A^	5.69 ± 0.21 ^A^	0.046 ± 0.002 ^E^	n.d.	0.046 ± 0.003 ^G^	6.56 ± 0.20 ^G^
*L.al.*	n.d.	n.d.	n.d.	3.35 ± 0.14 ^B^	n.d.	3.12 ± 0.06 ^A^	13.8 ± 0.9 ^D^
**Plants**	**QA Content (mg LE/g FL) ^a^**						
	**8**	**9**	**10**	**11**	**12**	**13**	
*L.po.*	1.08 ± 0.09 ^C^	3.54 ± 0.08 ^A^	n.d.	2.62 ± 0.09 ^A^	0.328 ± 0.013 ^C^	9.40 ± 0.75 ^A^	
*L.pe.*	0.735 ± 0.059 ^D^	0.043 ± 0.003 ^C^	0.048 ± 0.001 ^C^	0.068 ± 0.002 ^D^	0.154 ± 0.005 ^E^	4.09 ± 0.11 ^C^	
*L.b.*	5.41 ± 0.16 ^B^	n.d.	n.d.	n.d.	0.209 ± 0.017 ^D^	0.051 ± 0.005 ^F^	
*L.mu.*	n.d.	0.729 ± 0.008 ^B^	n.d.	0.727 ± 0.011 ^B^	0.812 ± 0.012 ^B^	4.98 ± 0.23 ^B^	
*L.mi.*	0.176 ± 0.011 ^E^	n.d.	n.d.	n.d.	0.045 ± 0.002 ^F^	0.063 ± 0.006 ^F^	
*L.arg.*	n.d.	n.d.	6.08 ± 0.07 ^A^	n.d.	4.88 ± 0.34 ^A^	n.d.	
*L.arb*	n.d.	n.d.	4.61 ± 0.13 ^B^	n.d.	n.d.	0.104 ± 0.008 ^E^	
*L.al.*	6.15 ± 0.08 ^A^	n.d.	n.d.	0.377 ± 0.011 ^C^	0.910 ± 0.017 ^B^	4.97 ± 0.21 ^B^	

**^a^** Contents expressed as mg of lupanine equivalents per gram of fresh leaves (mg LE/g FL); the presented values comprise means ± standard deviation (*n* = 10). *L.po. = L. polyphyllus*, *L.pe. = L. perennis*, *L.b. = L. bogotensis*, *L.mu. = L. mutabilis*, *L.mi. = L. mirabilis*, *L.arg = L. argenteus*, *L.arb. = L. arboreus*, *L.al. = L. albus*. Different uppercase capital letters indicate statistically significant differences according to the post hoc Tukey test (*p* < 0.05). n.d. = not detected.

**Table 3:** Contents of quinolizidines detected in extracts from *L. polyphyllus* leaves after pruning events (p1–p12).

PE ^a^	QA Content (mg LE/g FL) ^b^				
	1	4	5	6	7
**PE1**	0.011 ± 0.001 ^F^	0.123 ± 0.011 ^G^	1.73 ± 0.09 ^G^	1.07 ± 0.08 ^E^	11.3 ± 0.4 ^E^
**PE2**	0.009 ± 0.001 ^F^	0.115 ± 0.003 ^G^	2.77 ± 0.22 ^EF^	1.12 ± 0.09 ^E^	14.8 ± 0.4 ^C^
**PE3**	0.014 ± 0.001 ^F^	0.130 ± 0.009 ^FG^	1.42 ± 0.11 ^G^	1.70 ± 0.12 ^C^	12.7 ± 0.3 ^D^
**PE4**	0.016 ± 0.001 ^E^	0.269 ± 0.011 ^E^	3.57 ± 0.14 ^D^	0.90 ± 0.06 ^F^	13.7 ± 0.7 ^CD^
**PE5**	0.118 ± 0.006 ^D^	0.341 ± 0.017 ^D^	5.32 ± 0.08 ^B^	1.39 ± 0.04 ^D^	18.6 ± 0.6 ^A^
**PE6**	0.184 ± 0.015 ^C^	0.094 ± 0.003 ^G^	8.72 ± 0.09 ^A^	3.36 ± 0.22 ^B^	14.8 ± 0.8 ^C^
**PE7**	0.375 ± 0.004 ^A^	0.137 ± 0.001 ^F^	8.93 ± 0.17 ^A^	5.15 ± 0.10 ^A^	16.9 ± 0.7 ^B^
**PE8**	0.282 ± 0.006 ^B^	0.139 ± 0.003 ^F^	8.52 ± 0.15 ^A^	3.57 ± 0.21 ^B^	15.5 ± 0.5 ^C^
**PE9**	0.131 ± 0.007 ^D^	0.003 ± 0.001 ^H^	5.04 ± 0.21 ^B^	0.009 ± 0.001 ^G^	13.4 ± 0.8 ^CD^
**PE10**	tr.	1.602 ± 0.128 ^B^	4.49 ± 0.14 ^C^	1.12 ± 0.05 ^E^	11.7 ± 0.5 ^E^
**PE11**	tr.	1.360 ± 0.054 ^C^	2.91 ± 0.12 ^E^	1.67 ± 0.08 ^C^	13.2 ± 0.6 ^D^
**PE12**	n.d.	4.026 ± 0.081 ^A^	2.43 ± 0.15 ^F^	tr.	13.4 ± 0.7 ^CD^
**PE ^a^**	**QA Content (mg LE/g FL) ^b^**				
	**8**	**9**	**11**	**12**	**13**
**PE1**	tr.	tr.	n.d.	0.055 ± 0.002 ^B^	1.74 ± 0.10 ^F^
**PE2**	tr.	0.014 ± 0.001 ^F^	n.d.	0.076 ± 0.003 ^A^	1.86 ± 0.15 ^EF^
**PE3**	tr.	0.162 ± 0.008 ^B^	n.d.	tr.	1.42 ± 0.08 ^G^
**PE4**	tr.	0.132 ± 0.009 ^C^	n.d.	tr.	1.57 ± 0.09 ^FG^
**PE5**	0.068 ± 0.003 ^B^	0.051 ± 0.006 ^E^	n.d.	tr.	1.65 ± 0.13 ^F^
**PE6**	0.004 ± 0.001 ^C^	0.074 ± 0.005 ^D^	n.d.	n.d.	4.84 ± 0.21 ^B^
**PE7**	0.003 ± 0.001 ^C^	0.201 ± 0.011 ^A^	tr.	n.d.	14.7 ± 0.5 ^A^
**PE8**	0.149 ± 0.012 ^A^	0.088 ± 0.001 ^D^	tr.	n.d.	14.3 ± 0.4 ^A^
**PE9**	tr.	n.d.	tr.	n.d.	2.47 ± 0.19 ^D^
**PE10**	n.d.	n.d.	1.69 ± 0.07 ^A^	n.d.	2.10 ± 0.14 ^E^
**PE11**	n.d.	n.d.	1.52 ± 0.06 ^B^	n.d.	4.01 ± 0.18 ^C^
**PE12**	n.d.	n.d.	0.039 ± 0.005 ^C^	n.d.	4.53 ± 0.19 ^BC^

^a^ PE = pruning events, i.e., twelve time periods for cutting off leaves from the same living, growing *L. polyphyllus* plants (*n* = 10). ^b^ Contents expressed as mg of lupanine equivalents per gram of fresh leaves (mg LE/g FL); the presented values comprise means ± standard deviation (*n* = 10). Different uppercase capital letters indicate statistically significant differences according to the post hoc Tukey test (*p* < 0.05). n.d. = not detected. tr. = traces.

## Data Availability

The data that support the findings of this study are available from the corresponding author upon reasonable request.

## Supplementary Materials

The following supporting information can be downloaded online. Table S1: Yields of QREs obtained from propagated *Lupinus* species under greenhouse, Table S2: Replicates of the mycelial growth inhibition percentages of each lupin-based treatment, Figure S1: structures of QA identified in the eight species of *Lupinus*, Figure S2: Antifungal activity against *F. oxysporum* of eight *Lupinus* species, and Equation (S1): Retention index calculation.

## Sample Availability

Samples of the substances are available from the corresponding author upon reasonable request.
