# Supplementary material for: Quinolizidine-Based Variations and Antifungal Activity of Eight Lupinus Species Grown under Greenhouse Conditions
Source: Molecules. 2022 Jan 4;27(1):305. doi: 10.3390/molecules27010305 (PMC8746871; doi:10.3390/molecules27010305)
Supplement: Supplementary file 1 [file molecules-27-00305-s001.zip › molecules-1503788-supplementary.pdf]

Supplementary Material

Quinolizidine-Based Variations and Antifungal Activity of Eight Lupinus Species Grown under Greenhouse Conditions

Willy Cely-Veloza, Diego Quiroga Daza, Ericsson Coy-Barrera

Bioorganic Chemistry Laboratory, Department of Chemistry, Universidad Militar Nueva Granada, Cajicá 250247, Colombia.

Content

		Page
**Table S1**	Yields of QREs obtained from propagated Lupinus species under greenhouse	2
**Table S2**	Replicates of the mycelial growth inhibition percentages of each lupin-based treatment.	2
**Figure S1**	Structures of QA identified in the eight species of Lupinus	3
**Figure S2**	Antifungal activity against *F. oxysporum* of eight Lupinus species.	3
**Equation S1**	Retention index calculation	4

Table S1. Yields of QREs obtained from propagated Lupinus species under greenhouse.

Plant	fresh leaves (g)	QRE (g)	Yield (%)
Lupinus polyphyllus	5.1922 ± 0.3531^{A}	0.2371 ± 0.0166^{B}	4.566 ± 0.230^{B}
Lupinus perennis	5.1681 ± 0.2481^{A}	0.2352 ± 0.0157^{B}	4.551 ± 0.523^{B}
Lupinus bogotensis	5.1095 ± 0.3168^{A}	0.2834 ± 0.0192^{A}	5.547 ± 0.320^{A}
Lupinus mutabilis	5.0144 ± 0.3109^{A}	0.2753 ± 0.0146^{A}	5.490 ± 0.331^{A}
Lupinus mirabilis	5.0513 ± 0.3031^{A}	0.3052 ± 0.0122^{A}	6.042 ± 0.204^{A}
Lupinus argenteus	4.9245 ± 0.3250^{A}	0.1235 ± 0.0060^{C}	2.508 ± 0.188^{C}
Lupinus arboreus	5.0223 ± 0.2310^{A}	0.1392 ± 0.0067^{C}	2.772 ± 0.160^{C}
Lupinus albus	5.0764 ± 0.3401^{A}	0.2186 ± 0.0096^{B}	4.306 ± 0.277^{B}

Values expressed as means ± standard deviation (n = 10). Different uppercase capital letters indicate statistically significant differences according to the post hoc Tukey test ($p < 0.05$).

Table S2. Replicates of the mycelial growth inhibition percentages of each lupin-based treatment.

Species	5.0 µg/µL			SD	1.0 µg/µL			SD	0.1 µg/µL			SD
Lb	92.8	90.2	89.1	1.90	80.0	80.2	80	0.36	68.1	70.2	74.2	3.1
Lmu	80.2	83.6	82.4	1.72	58.9	60.3	62	1.60	49.0	53.2	50.8	2.1
Lmi	92.0	88.4	90.6	1.81	86.0	80.4	84	2.80	72.5	70.6	70.1	1.3
Lar	60.3	65.4	70.1	4.90	39.9	44.2	44	2.39	24.4	27.4	28.7	2.2
Lab	57.6	60.3	58.4	1.38	45.0	40.3	42	2.39	37.9	40.5	36.8	1.9
Lal	50.7	52.9	53.6	1.49	38.4	40.3	40	0.95	25.8	27.5	26.2	0.9
Lpe	67.7	70.2	69.4	1.29	56.1	57.3	51	3.40	34.1	31.6	36.9	2.7
Lpo	94.6	92.1	93.5	1.26	93.5	90.2	95	2.63	86.9	83.1	86.3	2.0
**M47**	97.1	95.6	94.9	1.12	93.0	95.6	95	1.31	90.0	94.6	91.8	2.3
**R**	98.3	97.9	98.1	0.20	95.0	96.5	97	1.12	94.0	95.9	97.1	1.6

R = rovral, **M47** = dithane, ***Lpo*** = *L. polyphyllus*, ***Lb*** = *L. bogotensis*, ***Lmi*** = *L. mirabilis*, ***Lmu*** = *L. mutabilis*, ***Lpe*** **=** *L. perennis*, ***Lab*** **=** *L. arboreus*, ***Lal*** = *L. albus*, ***Lar***= *L. argenteus*, **SD** = Standard deviation

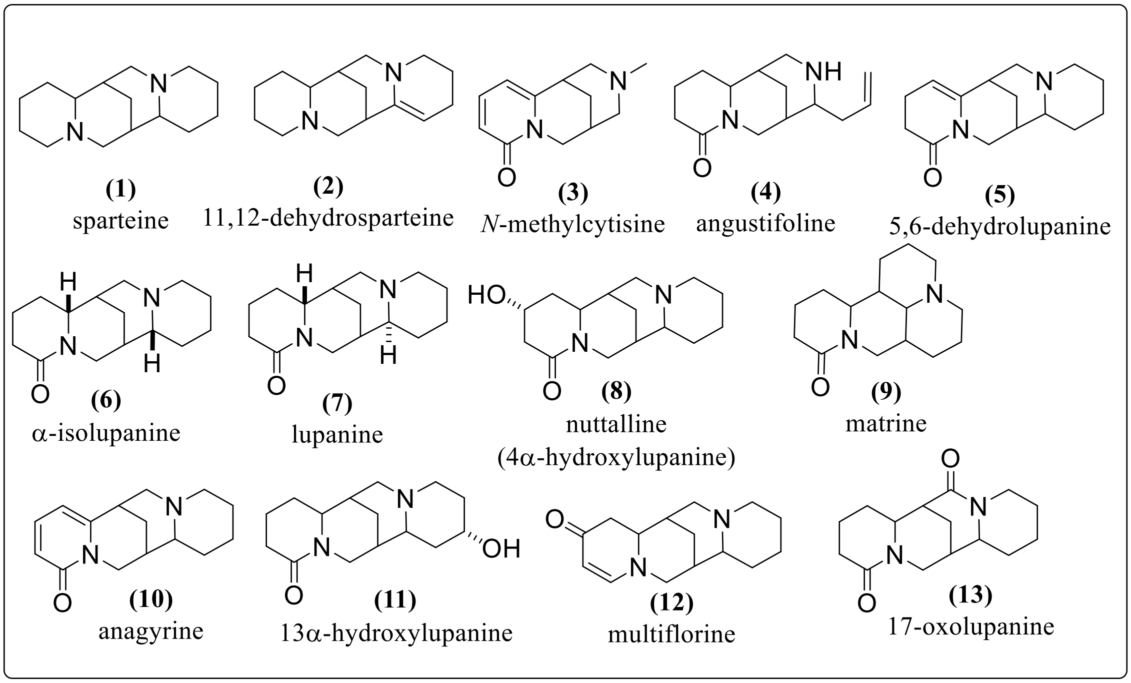

Figure S1. Structures of QA identified in the eight species of Lupinus

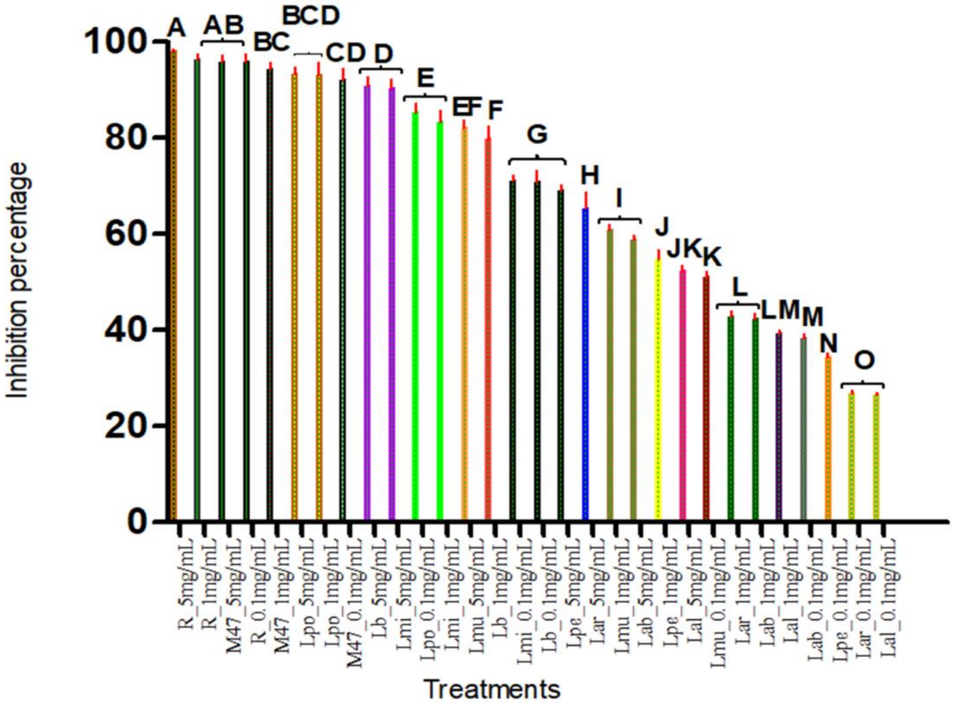

Figure S2. Antifungal activity against *F. oxysporum* of eight Lupinus species. Significantly different groups obtained by Tukey's statistical test. (**R** = rovral, **M47** = dithane, **Lpo** = *L. polyphyllus*, **Lb** = *L. bogotensis*, **Lmi** = *L. mirabilis*, **Lmu** = *L. mutabilis*, **Lpe =** *L. perennis*, **Lab =** *L. arboreus*, **Lal** = *L. albus*, **Lar**= *L. argenteus*). Different letters indicated statistically different groups (p>0.05).

Equation S1. Retention Index (RI) calculation:

$$RI = 100n + 100\,[\log(t'_x) - \log(t'_n)] / [\log(t'_{n+1}) - \log(t'_n)]$$

where t'_n and t'_{n+1} are the adjusted retention times of the reference *n*-alkane hydrocarbons (C_{10}-C_{24}) that are eluted immediately before and after compound "X" and t'_x is the adjusted retention time of compound "X". The retention index (**RI**) of compound "X" represents one hundred times the number of carbon atoms in the molecule of a hypothetical hydrocarbon that has the same retention as compound "X" (Babushok, 2015).
